# Plasma bile acids show a positive correlation with body mass index and are negatively associated with cognitive restraint of eating in obese patients

**DOI:** 10.3389/fnins.2015.00199

**Published:** 2015-06-03

**Authors:** Philip Prinz, Tobias Hofmann, Anne Ahnis, Ulf Elbelt, Miriam Goebel-Stengel, Burghard F. Klapp, Matthias Rose, Andreas Stengel

**Affiliations:** ^1^Division of General Internal and Psychosomatic Medicine, Charité Center for Internal Medicine and Dermatology, Charité-Universitätsmedizin BerlinBerlin, Germany; ^2^Division for Endocrinology, Diabetes, and Nutrition, Charité Center for Internal Medicine with Gastroenterology and Nephrology, Charité-Universitätsmedizin BerlinBerlin, Germany; ^3^Department of Internal Medicine and Institute of Neurogastroenterology, Martin-Luther-KrankenhausBerlin, Germany

**Keywords:** anorexia, body mass index, eating behavior, obesity, physical activity, psychometric, stress

## Abstract

Bile acids may be involved in the regulation of food intake and energy metabolism. The aim of the study was to investigate the association of plasma bile acids with body mass index (BMI) and the possible involvement of circulating bile acids in the modulation of physical activity and eating behavior. Blood was obtained in a group of hospitalized patients with normal weight (BMI 18.5–25 kg/m^2^), underweight (anorexia nervosa, BMI < 17.5 kg/m^2^) and overweight (obesity with BMI 30–40, 40–50 and >50 kg/m^2^, *n* = 14–15/group) and plasma bile acid concentrations assessed. Physical activity and plasma bile acids were measured in a group of patients with anorexia nervosa (BMI 14.6 ± 0.3 kg/m^2^, *n* = 43). Lastly, in a population of obese patients (BMI 48.5 ± 0.9 kg/m^2^, *n* = 85), psychometric parameters related to disordered eating and plasma bile acids were assessed. Plasma bile acids showed a positive correlation with BMI (*r* = 0.26, *p* = 0.03) in the population of patients with broad range of BMI (9–85 kg/m^2^, *n* = 74). No associations were observed between plasma bile acids and different parameters of physical activity in anorexic patients (*p* > 0.05). Plasma bile acids were negatively correlated with cognitive restraint of eating (*r* = −0.30, *p* = 0.008), while no associations were observed with other psychometric eating behavior-related parameters (*p* > 0.05) in obese patients. In conclusion, these data may point toward a role of bile acids in the regulation of body weight. Since plasma bile acids are negatively correlated with the cognitive restraint of eating in obese patients, this may represent a compensatory adaptation to prevent further overeating.

## Introduction

Obesity is a growing health problem and predominantly responsible for morbidity and mortality (Ogden et al., 2006; James, 2008) due to its associated diseases such as type 2 diabetes mellitus, dyslipidemia and arteriosclerosis (Hevener and Febbraio, 2010) but also gastrointestinal diseases including certain forms of cancer, namely hepatocellular, colorectal, and pancreatic cancer (Calle et al., 2003). The prevalence continues to rise (Finucane et al., 2011) and is expected to reach 700 million obese people worldwide in 2015 (James, 2008). Therefore, the term “globesity” was introduced by the world health organization.

While treatment options—especially drug treatment—are limited so far (Bray, 2014), a better understanding of the regulation of hunger and satiety, energy expenditure and body weight is necessary. Hunger and satiety are predominantly regulated by peptidergic hormones (Hussain and Bloom, 2013), most of which are derived from the gastrointestinal tract (Suzuki et al., 2012). These encompass—among others—ghrelin, NUCB2/nesfatin-1 (Stengel and Taché, 2012), cholecystokinin, oxyntomodulin, pancreatic polypeptide, glucagon-like peptide 1 (GLP-1), and peptide YY (PYY) (Suzuki et al., 2012).

However, also other factors, particularly bile acids, have been hypothesized to be involved in the regulation of food intake. Since 95% of bile acids undergo enterohepatic circulation and are reabsorbed in the terminal ileum reaching the liver *via* the systemic circulation, bile acid levels can be assessed in stool as well as in blood (plasma, serum). Early on, an effect of bile acids on appetite has been described with a decrease following oral application of bile acids in obese patients (Bray and Gallagher, 1968), although the mechanisms involved remained unclear. More recently, an interaction of bile acids and food regulatory hormones has been identified. Rectal administration of taurocholic acid was reported to increase circulating levels of GLP-1 and PYY in healthy volunteers which may lead to the observed increase of fullness (Wu et al., 2013). This is likely to be a physiological effect as endogenous taurochenodeoxycholic acid showed positive correlations with the anorexigenic hormones GLP-1 and PYY, while a negative association with the orexigenic ghrelin was observed in healthy human subjects (Roberts et al., 2011). However, the possible role of bile acids in the modulation of eating behavior is yet to be further investigated.

The regulation of bile acids has been first studied under conditions of obesity indicating a blunted postprandial response of bile acids which could lead to a reduced anorexigenic signaling under these conditions (Glicksman et al., 2010). However, it is unclear whether bile acid levels are *per se* altered under conditions of chronically altered body weight.

Since physical activity—besides food intake—is a major determinant of body weight, a possible association of bile acids with physical activity has been investigated in few studies. One group reported reduced fecal bile acid levels in subjects with higher recreational physical activity compared to subjects of the lowest quartile of recreational physical activity (Wertheim et al., 2009). However, an animal study showed increased fecal bile acid levels in voluntarily wheel running mice compared to sedentary controls (Meissner et al., 2011). Whether this merely reflects species differences or whether other factors are important remains to be established.

The aim of the present study was to investigate a possible association of circulating bile acids with body mass index (BMI) over a broad range (BMI 9–85 kg/m^2^) in order to describe a modulation/adaptation under these conditions. Possible confounding factors such as cholesterol levels, statin use, and parameters of glucose control were assessed as well. Moreover, we studied a possible association of plasma bile acids and physical activity in a population of patients with anorexia nervosa, a disease that is often associated with significantly increased physical activity (Davis et al., 1997). Lastly, we also investigated a possible association of total plasma bile acids and eating behavior in obese patients with a broad range of disordered eating using well established questionnaires, namely the Eating Disorder Inventory (Garner et al., 1983; Paul and Thiel, 2004) and the Three-Factor Eating Questionnaire (Stunkard and Messick, 1985; Pudel and Westerhofer, 1989). Since obesity is often accompanied by other psychological disturbances (Gariepy et al., 2010; Luppino et al., 2010), we also assessed perceived stress, depressiveness, and anxiety.

## Materials and methods

### Subjects

All patients were hospitalized in the Division of Psychosomatic Medicine at Charité-Universitätsmedizin Berlin and gave written informed consent. Blood collection was performed on day 1, 2, or 3 after hospital admission before the onset of changes due to dietary treatment to increase or reduce body weight, respectively. All parameters were assessed on the same morning. The protocol was approved by the local ethics committee for human research (ethics committee Charité—Universitätsmedizin Berlin, Campus Charité Mitte, protocol number EA1/114/10).

For the investigation of a possible association of circulating bile acids with body weight, a total of 75 subjects (a) participated in this study and were divided into five groups: normal weight (BMI 18.5–25 kg/m^2^, 8 female, 7 male, age 21–61), underweight (anorexia nervosa, BMI < 17.5 kg/m^2^, 15 female, age 18–44) and overweight (different stages of obesity: BMI 30–40 kg/m^2^, age 21–61; BMI 40–50 kg/m^2^, age 20–68; and BMI > 50 kg/m^2^, age 28–73; *n* = 15/group with 8 female, 7 male/group). One patient took ursodesoxycholic acid and was therefore excluded from the analysis resulting in a final *n* = 74. The anthropometric characteristics are indicated in Table 1.

**Table 1:** **Anthropometric characteristics of patient population with broad range of body mass index**.

**Group**	**Body weight (kg)**		**BMI (kg/m^2^)**	
	**Mean ± SEM**	**Range**	**Mean ± SEM**	**Range**
Anorexia nervosa (15f)	35.9 ± 1.2	29.3–42.2^†^	13.1 ± 0.5	8.9–15.6
Normal weight (8f, 7m)	66.5 ± 2.7	53.3–79.9^†^	22.1 ± 0.3	20.3–25.0
Obesity BMI 30–40 (8f, 7m)	113.7 ± 3.2	91.4–140.6^†^	37.6 ± 0.7	30.1–39.8
Obesity BMI 40–50 (8f, 6m)	135.5 ± 3.8	113.5–161.0^†^	45.6 ± 0.8	41.1–49.7
Obesity BMI > 50 (8f, 7m)	194.4 ± 7.9	151.8–261.9^†^	67.1 ± 1.8	59.0–84.6

N = 14–15/group. Distribution of the data was determined by using the Kolmogorov-Smirnov test.

† *Indicates normal distribution. BMI, body mass index; f, female; m, male*.

In another cohort (b) of 43 female anorexic patients (age 18–52) the possible association of circulating bile acids with physical activity was investigated. The anthropometric characteristics are indicated in **Table 3**.

Lastly, a population of 85 female obese patients (c, age 19–73) was investigated to study the association of plasma bile acids with psychometric parameters involved in eating behavior. The anthropometric characteristics are indicated in **Table 5**.

All subjects were devoid of a history for gastrointestinal surgery. Exclusion criteria also encompassed an age < 18 years, current pregnancy and non-treated psychotic diseases. All normal weight patients were hospitalized exclusively due to psychosomatic disorders with functional bodily symptoms (functional gastrointestinal symptoms excluded) without relevant somatic disorders. Anorexic and obese patients were diagnosed according to the International Classification of Diseases-10 (ICD-10) and hospitalized for weight gain or weight loss therapy, respectively. In obese patients hypercortisolism was excluded by assessment of urinary free cortisol excretion in a 24 h sample or—in case of clinical suspicion—with an overnight low-dose dexamethasone suppression test.

### Blood collection

After an overnight fast, venous blood was withdrawn between 07:00 and 08:00 a.m. in cooled EDTA tubes containing aprotinin (0.6 trypsin inhibitor/0.5 ml blood; ICN Pharmaceuticals, Costa Mesa, CA, USA) and immediately centrifuged at 3000 rpm for 10 min at 4°C. Plasma was separated and aliquots stored at −80°C until further processing. Patients were advised not to smoke or exercise that morning.

### Measurements

#### Bile acid concentration

Plasma levels of total bile acids were determined using a commercial total bile acid assay kit (# BQ 042A-EALD, BQ Kits Inc., San Diego, CA, USA). The enzyme 3-α-hydroxysteroid dehydrogenase oxidates bile acids to 3-keto steroids in the presence of Thio-NAD which is reduced to Thio-NADH. The absorbance of Thio-NADH can be quantitatively measured and is used to indirectly determine the levels of total bile acids. Samples were prepared according to the manufacturer's protocol, incubated for 3 min at 37°C and the absorbance was measured at 405 nm 1 and 2 min after incubation. All samples were processed in one batch. The intra-assay variability was 9.3%.

#### Blood glucose, serum insulin, triglycerides, and cholesterol

Parameters such as blood glucose and serum insulin, triglycerides, and cholesterol were analyzed using the routine laboratory analyses (Charité-Universitätsmedizin Berlin).

#### Oral glucose tolerance test

Overnight fasted patients received a standardized oral glucose solution (75 g) and blood was withdrawn before and at 1–2 h post ingestion to analyze blood glucose and serum insulin levels.

#### Body weight and body mass index

Body weight and height were assessed in overnight fasted subjects wearing underwear only. BMI was calculated as kg/m^2^.

#### Abdominal ultrasound

Patients underwent routine abdominal ultrasound examination to exclude or diagnose fatty liver disease.

#### Body composition

Body composition was estimated using bioelectrical impedance analysis (BIA) which was performed on the day of the blood withdrawal between 10:30 a.m. and 01:00 p.m. after a fasting period of at least 2 h as described before (Stengel et al., 2013). Briefly, BIA was performed under standardized conditions (patient in supine position, arms relaxed at the sides without touching the body, and thighs separated) using a bioelectrical impedance analyzer (Nutrigard-M, Data Input, Darmstadt, Germany) and corresponding electrodes (Bianostic-AT, Data Input, Germany). Before bioimpedance testing, fasted patients voided, and had an equilibration period of 30 min in the supine position. Total body water (TBW), fat free mass (FFM), extracellular mass (ECM), body cell mass (BCM, best reflecting muscle mass and muscle containing compartments of the body) and fat mass (FM) were obtained using the equations provided by the manufacturer's software (NutriPlus, Data Input, Germany).

#### Physical activity

Physical activity was continuously measured for 3 days during inpatient treatment around the time of the blood withdrawal using a portable armband device (SenseWear™ PRO3 armband; BodyMedia Inc., Pittsburgh, PA, USA) as recently described (Hofmann et al., 2014). Briefly, 1 day was included in the data analysis if the minimum duration of data acquisition was 20 h and 30 min per day. Patients were not activity restricted during the measurement. Although activity patterns under conditions of hospitalization may differ from those in daily life, activity habits are likely to persist during inpatient treatment if activity is not restricted due to a genetic influence on physical activity (Joosen et al., 2005). The SenseWear™ armband device uses a multisensory array including sensors measuring heat flux, galvanic skin response, skin temperature, near-body ambient temperature and a 2-axis accelerometer. Step counts were measured by the accelerometer and directly taken for analyses. Obtained data were analyzed using a generalized proprietary algorithm developed by the manufacturer (SenseWear™ Software, Version 6.1, BodyMedia Inc.).

Total energy expenditure (TEE) consists of resting energy expenditure (REE), thermic effect of food (TEF) and activity thermogenesis. Activity thermogenesis can be further separated into two components: exercise-related activity thermogenesis (EAT, deliberate physical exercise, sports) and non-exercise activity thermogenesis (NEAT, spontaneous daily physical activity) (Levine et al., 1999). Energy expenditure of more than five metabolic equivalents of a task (METs, a physiological measure expressing the energy cost of physical activities) was classified as EAT, whereas energy expenditure of up to five METs was classified as NEAT as described before (Ainsworth et al., 2000; Elbelt et al., 2010). While TEE, exercise activity thermogenesis (EAT) and duration of exercise were directly received from the proprietary algorithms of the SenseWear™ armband device, NEAT was calculated according to the equation: NEAT = TEE–thermic effect of food (TEF)–REE–EAT. TEF was estimated as 10% of TEE and calculated as TEE × 0.1 (Levine, 2002). REE, required for calculation of NEAT, was calculated using weight-specific REE prediction equations (Muller et al., 2004) since REE cannot be directly determined by the SenseWear™ armband device.

#### Psychometric parameters

Psychometric parameters were assessed on the day of the blood withdrawal using an electronic handheld device.

The Three-Factor Eating Questionnaire (TFEQ) (Stunkard and Messick, 1985) employs 66 items to assess eating behavior on 3 scales: “cognitive restraint of eating,” “disinhibition,” and “hunger.” We used the German version (Pudel and Westerhofer, 1989).

The Eating Disorder Inventory (EDI) (Garner et al., 1983) was developed to assess symptoms of disordered eating in anorexic and bulimic patients encompassing 64 items on 8 subscales, namely “drive for thinness,” “bulimia,” “body dissatisfaction,” “ineffectiveness,” “perfectionism,” “interpersonal distrust,” “interoceptive awareness,” and “maturity fears.” We used the German version (Thiel et al., 1997) of the 2nd version (Garner, 1991) which added three subscales to the original one, but only used the first eight subscales of the EDI-2 mentioned above. Sum scores of all scales were converted into percentage values ranging from 0 to 100.

Two scales of the patient health questionnaire (PHQ) (Spitzer et al., 1999) were carried out to assess general anxiety (GAD-7) and depression (PHQ-9). The German version of the GAD-7 (Lowe et al., 2008), a 7-item scale was primarily designed to diagnose general anxiety disorder (Spitzer et al., 2006) but is also sensitive as a screening tool for panic, social anxiety and posttraumatic stress disorders. The widely used PHQ-9 depression scale (Spitzer et al., 1999) is a 9-item screening instrument for the diagnosis of major depression and the evaluation of severity of depressive symptoms which was used in the German version (Löwe et al., 2002).

The perceived stress questionnaire (PSQ) (Levenstein et al., 1993) used in its revised German 20-item version (PSQ-20) (Fliege et al., 2005) is an instrument based on a stress concept that emphasizes the subjective experience and—besides an overall score—allows to evaluate the four subscales “worries,” “tension,” “joy” as stress responses and “demands” as the perception of external stressors. Sum scores of all scales were converted into percentage values ranging from 0 to 100.

### Statistical analysis

Distribution of the data was determined by using the Kolmogorov-Smirnov test. Data are expressed as mean ± standard error of the mean (SEM). Differences between two groups were assessed using the *t*-test, differences between more groups were calculated using One-Way analysis of variance (ANOVA) followed by Tukey *post-hoc* test (study population a). Correlations were determined by Pearson's or Spearman's analysis depending on the distribution of the data (study populations a, b, and c). Differences between groups were considered significant when *p* < 0.05. All statistical analyses were conducted using SigmaStat 3.1 (Systat Software, San Jose, CA, USA).

## Results

### Plasma bile acids show a positive correlation with body mass index

**Figure 1:**
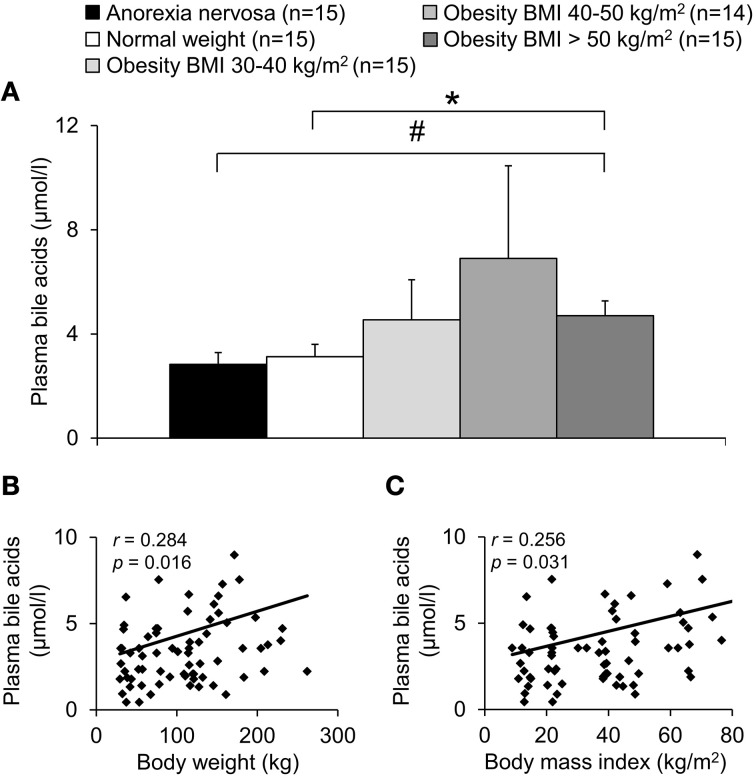
**Plasma bile acids show a positive association with body weight**. The study population was divided into five groups: underweight (anorexia nervosa), normal weight, and overweight (obese subjects with BMI between 30–40, 40–50, and > 50 kg/m^2^). The plasma concentration of bile acids shows an increase with BMI reaching significance in the obese group with BMI > 50 kg/m^2^ compared to the anorexic and normal weight group **(A)**. A positive correlation is observed between plasma bile acids and body weight **(B)** as well as BMI **(C)**. Values for *r* and *p* are indicated in the correlation graphs. Data in **(A)** are expressed as mean ± sem of *n* = 14–15 subjects/group. ^*^p < 0.05 vs. normal weight and ^#^*p* < 0.05 vs. anorexia nervosa.

A population of 74 subjects with a broad range of BMI (8.9–84.6 kg/m^2^, cohort a) was divided into five groups depending on their BMI (Table 1). Total plasma bile acids were similar in patients with normal weight and anorexia nervosa (*p* = 0.66) but showed a gradual increase with increasing BMI with highest levels in the obese group with BMI 40–50 kg/m^2^ (+121% compared to normal weight group, Figure 1A). However, due to the high variation in the BMI group 40–50 kg/m^2^ significance for plasma bile acids was reached in the BMI > 50 kg/m^2^ group only compared to normal weight (+51%, *p* = 0.04) and anorexia nervosa (+66%, *p* = 0.02; Figure 1A). Analysis of all five BMI groups showed a positive correlation of total plasma bile acid levels and body weight (*r* = 0.28, *p* = 0.02; Figure 1B) as well as BMI (*r* = 0.26, *p* = 0.03; Figure 1C).

While in the group of anorexia nervosa only female subjects were included, all other groups contained female as well as male subjects (Table 1). No sex differences were observed for total plasma bile acid levels in female vs. male subjects (*p* = 0.17; data not shown). Lastly, no correlation of bile acids and age was observed (*r* = −0.12, *p* = 0.32; data not shown).

### Plasma bile acids are not associated with circulating glucose or cholesterol levels

To exclude possible confounding factors we analyzed the population of 74 subjects with broad range of BMI also with regards to comorbidities (Table 2). No correlation was observed between fasting circulating glucose and bile acid levels (*r* = 0.09, *p* = 0.47; data not shown). In addition, no differences in fasting total plasma bile acid levels in obese patients with or without type 2 diabetes were detected (population of patients with broad range of BMI: *n* = 44 obese, *n* = 13 with type 2 diabetes mellitus, *n* = 31 without, *p* = 0.18 for comparison of plasma bile acids between both groups; for BMI *p* = 0.65; data not shown). Moreover, no correlation was observed between circulating cholesterol and plasma bile acid levels (*r* = 0.13, *p* = 0.27; data not shown). Lastly, after exclusion of patients with arterial hypertension (*r* = 0.32, *p* = 0.03), fatty liver disease (*r* = 0.28, *p* = 0.04) or psychopharmacological treatment (*r* = 0.28, *p* = 0.03), the positive association of bile acids and BMI remained visible (data not shown).

**Table 2:** **Comorbidities and medication of the three study populations (a, b, and c)**.

**Parameter**	**Broad range of body mass index (*n* = 74)**					**Broad activity pattern (*n* = 43)**	**Broad range of disordered eating (*n* = 85)**
	**Anorexia nervosa (*n* = 15)**	**Normal weight (*n* = 15)**	**Obesity BMI 30–40 kg/m^2^ (*n* = 15)**	**Obesity BMI 40–50 kg/m^2^ (*n* = 14)**	**Obesity BMI > 50 kg/m^2^ (*n* = 15)**		
**COMORBIDITIES**							
Type 2 diabetes mellitus	0 (0.0%)	0 (0.0%)	4 (26.7%)	3 (21.4%)	6 (40.0%)	0 (0.0%)	19 (22.4%)
Impaired fasting glucose	n.t.	n.t.	0 (0.0%)	1 (7.1%)	0 (0.0%)	n.t.	4 (4.7%)
Insulin resistance with normal glucose control	n.t.	n.t.	0 (0.0%)	3 (21.4%)	3 (20.0%)	n.t.	22 (25.9%)
Arterial hypertension	0 (0.0%)	2 (13.3%)	7 (46.7%)	9 (64.3%)	10 (66.7%)	0 (0.0%)	40 (47.1%)
Hypercholesterinemia	1 (6.7%)	1 (6.7%)	5 (33.3%)	5 (35.7%)	9 (60.0%)	8 (18.6%)	39 (45.9%)
Hypertriglyceridemia	0 (0.0%)	0 (0.0%)	2 (13.3%)	3 (21.4%)	4 (26.7%)	0 (0.0%)	16 (18.8%)
Hyperuricemia	0 (0.0%)	0 (0.0%)	2 (13.3%)	4 (28.6%)	9 (60.0%)	1 (2.3%)	32 (37.6%)
Fatty liver disease	0 (0.0%)	0 (0.0%)	9 (60.0%)	9 (64.3%)	3 (20.0%)	0 (0.0%)	48 (56.5%)
**MEDICATION**							
Oral antidiabetics	0 (0.0%)	0 (0.0%)	1 (6.7%)	1 (7.1%)	2 (13.3%)	0 (0.0%)	10 (11.8%)
Insulin	0 (0.0%)	0 (0.0%)	1 (6.7%)	1 (7.1%)	0 (0.0%)	0 (0.0%)	3 (3.5%)
Statins	0 (0.0%)	0 (0.0%)	1 (6.7%)	0 (0.0%)	4 (26.7%)	0 (0.0%)	11 (12.9%)
Psychopharmacologicals	3 (20.0%)	2 (13.3%)	4 (26.7%)	1 (7.1%)	3 (20.0%)	7 (16.3%)	27 (31.8%)

*Values are given in total numbers (% in parentheses). AN, anorexia nervosa; BMI, body mass index; n.t., not tested*.

### Plasma bile acids are not associated with parameters of physical activity and body composition in patients with anorexia nervosa

**Figure 2:**
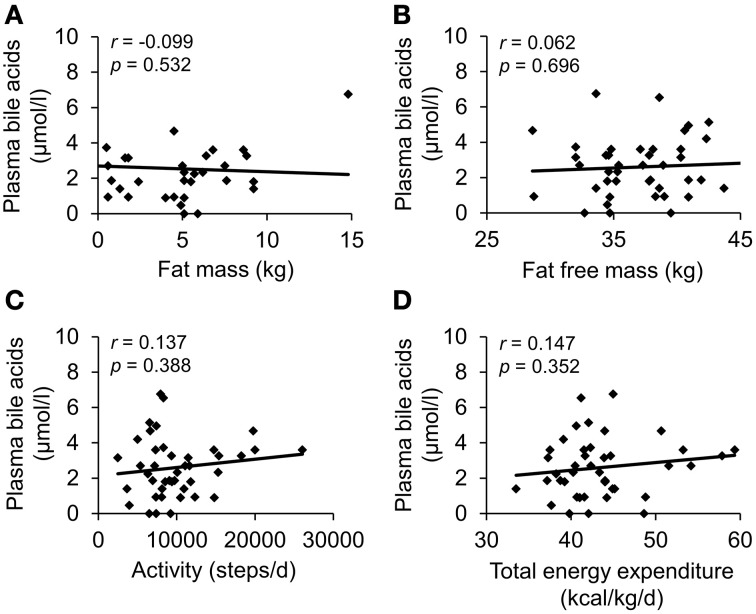
**Plasma bile acids do not show a correlation with body composition or energy expenditure in patients with anorexia nervosa**. The study population consisted of 43 anorexic subjects with broad activity pattern. Body composition was assessed using bioelectrical impedance analysis; physical activity was monitored with a portable armband device. No correlation was observed between plasma bile acids and fat mass **(A)**, fat free mass **(B)**, physical activity expressed as steps/d **(C)** and total energy expenditure **(D)**. Values for *r* and *p* are indicated in the correlation graphs.

To investigate a possible association of circulating bile acids with physical activity a population of patients with anorexia nervosa with broad activity pattern (cohort b) was included (*n* = 43, steps/day: 2479–26047, Table 3). Total plasma bile acids did not show any correlation with parameters of body composition or physical activity (Figure 2, Table 4).

**Table 3:** **Anthropometric characteristics, body composition, and physical activity parameters in a population of anorexic patients with broad activity pattern**.

**Parameter**	**Mean ± SEM**	**Range**
**ANTHROPOMETRIC CHARACTERISTICS**		
Body weight (kg)	40.3 ± 1.0	28.1–52.0^†^
Body mass index (kg/m^2^)	14.6 ± 0.3	10.8–17.7^†^
**BODY COMPOSITION**		
Fat mass (kg)	2.8 ± 0.8	0.0–14.8^‡^
Body cell mass (kg)	15.9 ± 0.4	8.4–21.4^†^
Fat free mass (kg)	37.2 ± 0.6	28.6–45.1^†^
Extracellular mass (kg)	21.3 ± 0.6	15.4–33.9^‡^
Total body water (l)	27.2 ± 0.4	20.9–33.0^†^
**PHYSICAL ACTIVITY**		
Steps/d	10112 ± 728	2479–26047^‡^
Metabolic equivalents/d	1.8 ± 0.0	1.4–2.5^‡^
Total energy expenditure (kcal/kg/d)	43.5 ± 0.8	33.5–59.3^‡^
Resting energy expenditure (kcal/kg/d)	17.9 ± 0.2	14.8–19.5^‡^
Duration of exercise (min/d)	21.8 ± 11.0	0.0–471.0^‡^
Exercise activity thermogenesis (kcal/kg/d)	1.8 ± 0.8	0.0–31.7^‡^
Non exercise activity thermogenesis (kcal/kg/d)	19.5 ± 0.7	1.8–29.1^‡^

N = 43, all female. Distribution of the data was determined by using the Kolmogorov-Smirnov test.

† *indicates normal distribution*,

‡ *non-normal distribution*.

**Table 4:** **Correlation of plasma bile acids with physical activity in a population of anorexic patients with broad activity pattern**.

**Parameter**	***r***	***p***
**BODY COMPOSITION**		
Fat mass (kg)	−0.099	0.532
Body cell mass (kg)	−0.188	0.233
Fat free mass (kg)	0.062	0.696
Extracellular mass (kg)	0.193	0.220
Total body water (l)	0.058	0.715
**PHYSICAL ACTIVITY**		
Steps/d	0.137	0.388
Metabolic equivalents/d	0.162	0.304
Total energy expenditure (kcal/kg/d)	0.147	0.352
Resting energy expenditure (kcal/kg/d)	0.043	0.788
Duration of exercise (min/d)	0.090	0.570
Exercise activity thermogenesis (kcal/kg/d)	0.104	0.512
Non exercise activity thermogenesis (kcal/kg/d)	0.035	0.825

*N = 43, all female*.

### Plasma bile acids show a negative association with cognitive restraint of eating

**Figure 3:**
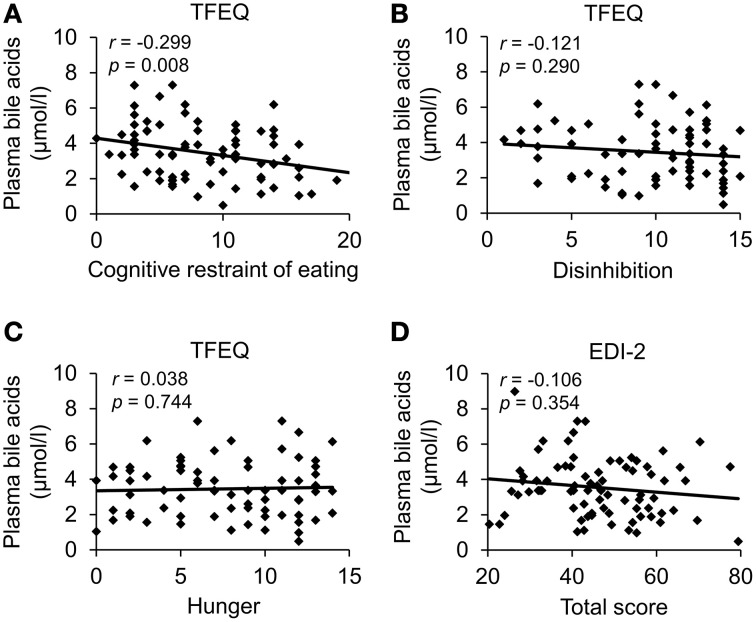
**Plasma bile acids show a negative association with cognitive restraint of eating in obese patients**. A population of obese patients (*n* = 85) with broad spectrum of disordered eating was investigated. Eating behavior was assessed using the Three-Factor Eating Questionnaire and the Eating Disorder Inventory. Plasma bile acids were negatively correlated with cognitive restraint of eating **(A)**, while no association was observed with disinhibition **(B)**, hunger **(C)** and the EDI-2 total score **(D)**. Values for *r* and *p* are indicated in the correlation graphs. Abbreviations: EDI-2, eating disorder inventory; TFEQ, Three-Factor Eating Questionnaire.

Next, we investigated a possible association of plasma bile acids with psychometric parameters in a population of obese patients with broad spectrum of disordered eating (cohort c, *n* = 85, EDI-2 total score: 20.4–79.4, Table 5). Total plasma bile acids showed a negative correlation with cognitive restraint of eating (*r* = −0.30, *p* = 0.01), while no correlation was observed with disinhibition (*r* = −0.12, *p* = 0.29) and hunger (TFEQ; *r* = 0.04, *p* = 0.74; Figure 3; Table 6). Similarly, no correlation of plasma bile acids was detected with the total score (Figure 3) or subscales (“drive for thinness,” “bulimia,” “body dissatisfaction,” “ineffectiveness,” “perfectionism,” “interpersonal distrust,” “interoceptive awareness,” and “maturity fears”) of the EDI-2 questionnaire (Table 6).

**Table 5:** **Anthropometric and psychometric characteristics in a population of obese patients with broad spectrum of disordered eating and depressiveness**.

**Parameter**	**Mean ± SEM**	**Range**
**ANTHROPOMETRIC CHARACTERISTICS**		
Body mass index (kg/m^2^)	48.5 ± 0.9	31.8–68.7^†^
**EATING HABITS**		
TFEQ		
- Cognitive restraint	8.5 ± 0.5	0–21^‡^
- Disinhibition	9.6 ± 0.4	1–15^‡^
- Hunger	7.7 ± 0.5	0–14^‡^
EDI total	46.2 ± 1.4	20–79^†^
- Drive for thinness	27.9 ± 0.8	11–41^†^
- Bulimia	17.5 ± 0.8	7–37^‡^
- Body dissatisfaction	49.5 ± 0.7	24–54^‡^
- Ineffectiveness	31.0 ± 1.1	15–54^†^
- Perfectionism	18.5 ± 0.7	7–34^†^
- Interpersonal distrust	23.7 ± 0.7	10–38^†^
- Interoceptive awareness	28.4 ± 0.9	10–55^†^
- Maturity fears	24.8 ± 0.8	11–44^†^
**DEPRESSIVENESS**		
PHQ-9	10.0 ± 0.7	0–25^†^
**ANXIETY**		
GAD-7	9.3 ± 0.6	0–21^‡^
**STRESS**		
PSQ total	54.9 ± 2.4	5–98^†^
- Worries	53.3 ± 3.0	0–100^‡^
- Tension	58.4 ± 2.9	0–100^†^
- Joy	40.6 ± 2.5	0–100^‡^
- Demands	48.7 ± 2.8	0–100^†^

N = 85, all female. Distribution of the data was determined by using the Kolmogorov-Smirnov test.

† *indicates normal distribution*,

‡ non-normal distribution. EDI, eating disorder inventory; GAD-7, Generalized Anxiety Disorder questionnaire; PHQ-9, patient health questionnaire; PSQ-20, perceived stress questionnaire 20-item scale; TFEQ, Three-Factor Eating Questionnaire.

**Table 6:** **Correlation of plasma bile acids with psychometric parameters in a population of obese patients with broad spectrum of disordered eating, depressiveness, anxiety, and stress**.

**Parameter**	***r***	***p***
**EATING HABITS**		
TFEQ		
- Cognitive restraint of eating	−**0.300**	**0.008**
- Disinhibition	−0.121	0.290
- Hunger	0.038	0.744
EDI total	−0.106	0.354
- Drive for thinness	−0.103	0.368
- Bulimia	−0.058	0.617
- Body dissatisfaction	−0.076	0.507
- Ineffectiveness	−0.122	0.289
- Perfectionism	−0.116	0.312
- Interpersonal distrust	−0.011	0.922
- Interoceptive awareness	0.024	0.835
- Maturity fears	−0.041	0.720
**DEPRESSIVENESS**		
PHQ-9	−0.128	0.256
**ANXIETY**		
GAD-7	−0.114	0.309
**STRESS**		
PSQ-20 total	−0.171	0.127
- Worries	−0.196	0.080
- Tension	−0.095	0.399
- Joy	0.152	0.175
- Demands	−0.131	0.244

*N = 85, all female. Significant correlations are displayed in bold. EDI, eating disorder inventory; GAD-7, Generalized Anxiety Disorder questionnaire; PHQ-9, patient health questionnaire; PSQ-20, perceived stress questionnaire 20-item scale; TFEQ, Three-Factor Eating Questionnaire*.

Since other psychological conditions may also affect eating behavior, we additionally investigated depressiveness, anxiety, and stress in the present population. However, no association was observed between total plasma bile acid levels and depressiveness (PHQ-9), anxiety (GAD-7) and stress (PSQ-20; Table 6).

## Discussion

In the present study we show that total plasma bile acids are higher in obese compared to normal weight and anorexic subjects which is reflected in a positive correlation with body mass index. These data are in line with an early study showing higher serum bile acid levels in overweight compared to ideal weight subjects (Halmy et al., 1986). The present study extends these findings by investigating the circulating bile acid levels over a very broad BMI spectrum (9–85 kg/m^2^). This increase is unlikely to depend on an increase in cholesterol levels as there was no association of circulating cholesterol and bile acid levels. It is to note that bile acids are not only produced by the liver but also by bacteria of the intestinal microbiota (Yoshimoto et al., 2013; Joyce et al., 2014). Since emerging evidence points toward greatly altered gut microbiota under conditions of obesity (Tilg et al., 2009; Deweerdt, 2014), this is likely to contribute to the altered plasma bile acid concentrations observed here.

Interestingly, besides this positive association, also the bile acid receptor, G protein-coupled bile acid receptor 1 (TGR5), expressed in white adipose tissue showed a positive correlation with body mass index (Svensson et al., 2013). Since this receptor is involved in the mediation of bile acid-induced release of GLP-1 (Thomas et al., 2009), enhanced signaling of this pathway on the level of the receptor and the ligand may result in increased anorexigenic signaling to prevent further overeating under conditions of obesity. On the other hand, higher levels of bile acids may also increase the risk for malignomas such as hepatocellular carcinoma (Yoshimoto et al., 2013), an effect that is likely related to the potential of deoxycholic acid to cause DNA damage (Kitazawa et al., 1990). This property has to be kept in mind when considering potential therapeutic implications of bile acids.

Bile acids have been investigated in the context of obesity-associated diseases, especially type 2 diabetes mellitus. Patients with type 2 diabetes mellitus displayed higher levels of circulating bile acids compared to healthy non-diabetic controls (Haeusler et al., 2013; Wewalka et al., 2014). This was also reflected in a positive correlation of circulating bile acids with insulin resistance in patients with and without type 2 diabetes (Cariou et al., 2011). However, another study described similar levels of baseline circulating bile acids in patients with type 2 diabetes and normoglycaemic controls (Vincent et al., 2013). Similarly, in the present study we did not detect any differences in fasting total plasma bile acid levels in obese patients with or without type 2 diabetes. However, these data have to be interpreted with caution as the group size of diabetic patients is small and only basal plasma levels of bile acids have been investigated, while differences may emerge under stimulated postprandial conditions as reported before (Vincent et al., 2013).

After describing the dependency of plasma bile acids with body mass index, we studied the possible association of circulating bile acids with body composition and physical activity in a population of anorexic subjects with widely differing levels of physical exercise. In this population no associations between plasma bile acids and different parameters of body composition, as assessed non-invasively by bioelectrical impedance analysis, were observed. In light of the positive association of bile acids with BMI observed in the present study, a recent study surprisingly described a negative association of body fat mass and bile acids in healthy normal weight subjects (Suzuki et al., 2014). These differences may be due to the investigation of fasting bile acid levels in the present vs. postprandial concentrations in the study of Suzuki and colleagues (Suzuki et al., 2014). Moreover, one has to keep in mind that the population of anorexic subjects might have too low amounts of body fat to study possible associations.

Anorexic patients showed a wide range of physical exercise behavior which was monitored using the SenseWear™ armband device used before in obese (Elbelt et al., 2010) and anorexic (Hofmann et al., 2014) patients. No associations were observed between levels of circulating bile acids and various markers of physical activity and energy expenditure. These data may indicate that plasma bile acids are not affected by physical activity, at least not under conditions of greatly reduced body weight. However, one previous study showed a reduction of fecal bile acid levels in subjects with higher recreational physical activity compared to lower recreational activity (Wertheim et al., 2009). These differences may be explained by the differences in the study sample (anorexic vs. overweight subjects), type of assessment (Sensewear™ vs. self-administered questionnaire) and most importantly the location of bile acid measurement (plasma vs. stool).

Nonetheless, bile acids are discussed to play a role in the mediation of energy expenditure. The TGR5 is not only expressed in white but also human brown adipose tissue (Svensson et al., 2013), a tissue crucially involved in non-shivering thermogenesis and energy expenditure (Cannon and Nedergaard, 2004; Cypess et al., 2009). Supporting the functional relevance of the receptor in this tissue, exogenous administration of bile acids increased energy expenditure in mice (Watanabe et al., 2006). This led to the hypothesis of using natural or semisynthetic ligands of the TGR5 in the drug treatment of obesity (Chen et al., 2011). In humans, expression of TGR5 showed a positive correlation with body weight as well as resting energy expenditure (Svensson et al., 2013), which might represent a physiological adaptation of the body to combat further body weight increase. Since regulation on the receptor level alone may not be sufficient, one would expect adaptation of the ligand as well. However, in line with the current study another group did not detect any correlation between circulating bile acids and energy expenditure in healthy or type 2 diabetic patients (Brufau et al., 2010). Further studies are warranted to investigate whether an increase (either endogenous or pharmacological) of circulating bile acids will stimulate energy expenditure and subsequently reduce body weight under conditions of obesity in humans.

Since—besides energy expenditure—food intake and related eating behavior is of utmost importance for body weight regulation especially under conditions of obesity, we next investigated a possible association of plasma bile acids with parameters of eating behavior in a population of obese subjects with a broad range of disordered eating. To exclude any possible sex differences only female subjects were investigated. Total plasma bile acids were negatively associated with the cognitive restraint of eating, while other parameters directly or secondarily related to eating behavior were not. One might speculate that in patients with lower cognitive restraint (patients were hospitalized for body weight reduction) the increase of plasma bile acids represents a mechanism to reduce food intake and body weight. Alternatively, since obese patients display higher bile acid levels, these individuals could also present with less cognitive restraint of eating without direct link to bile acids. Therefore, the possible connection has to be further investigated.

Despite the strength of the study of investigating study populations with broad ranges of the respective parameter of interest (BMI, activity, and disordered eating), several limitations should be acknowledged as well. In the present study we only assessed fasting bile acid levels. However, since food intake and especially food components affect the bile acid release also the postprandial bile acid response should be monitored. Ideally, also the diet should be controlled which was not possible in the present study. In addition, the groups studied were inhomogeneous with regards to comorbidities: e.g., diabetes mellitus type 2, hypercholesterinemia and statin treatment. Although no association between glucose or cholesterol levels and circulating bile acids was detected, subgroup analyses were not possible in the present study due to the sample size. Future studies should be performed with larger populations in order to allow for the analysis of adequately sized samples or after exclusion of patients with possibly confounding comorbidities. Moreover, the present study assessed total bile acid levels and therefore cannot comment on special subtypes of bile acids. Lastly, in order to further assess a cause-consequence relationship a longitudinal study should be performed assessing bile acids e.g., after weight loss in obese patients.

In summary, we show that plasma bile acids display a positive association with body mass index and may be involved in the regulation of body weight. No correlations were observed for circulating bile acids and parameters of physical activity and energy expenditure, at least not under conditions of greatly reduced body weight in patients with anorexia nervosa. Interestingly, total plasma bile acids show a negative association with the cognitive restraint of eating in obese patients with various levels of disordered eating, which may give rise to a compensatory biological mechanism to prevent further overeating and subsequently body weight gain in obese subjects.

## Author contributions

PP performed the laboratory analysis and reviewed the manuscript. TH recruited the patients and reviewed the manuscript. AA and UE contributed to the analysis of the data and critically reviewed the manuscript. MG-S helped to set up the method of bile acid analysis and gave critical input throughout the study. BK and MR were involved in planning the study and critically reviewed the manuscript. AS planned the study, analyzed the data and wrote the manuscript.

### Conflict of interest statement

The authors declare that the research was conducted in the absence of any commercial or financial relationships that could be construed as a potential conflict of interest.

## Abbreviations

BCM: body cell mass
BIA: bioelectrical impedance analysis
BMI: body mass index
EAT: exercise-related activity thermogenesis
ECM: extracellular mass
EDI: Eating disorder inventory
FFM: fat free mass
FM: fat mass
GAD-7: Generalized Anxiety Disorder 7-item scale
MET: metabolic equivalent of a task
NEAT: non-exercise activity thermogenesis
PHQ-9: Patient Health Questionnaire
PSQ-20: Perceived Stress Questionnaire 20-item scale
REE: resting energy expenditure
TBW: total body water
TEE: total energy expenditure
TEF: thermic effect of food
TFEQ: Three-Factor Eating Questionnaire.
